# A Multi-Omics Integration Analysis Reveals That *Mori Fructus* Polysaccharide Ameliorates Liver Injury via Regulating Liver Metabolic Function Through Inhibiting Lipid Metabolism, Enhancing Glycolysis, and Promoting Amino Acid Utilization

**DOI:** 10.3390/antiox15040443

**Published:** 2026-04-01

**Authors:** Qingfang Deng, Baitong Jing, Ruhai Chen, Yang Cao, Xiaomei Zhou, Yu Sun, Xin Zhou

**Affiliations:** 1 Key Laboratory for Information System of Mountainous Areas and Protection of Ecological Environment, Guizhou Normal University, Guiyang 550025, China; 201510002@gznu.edu.cn (Q.D.); j13634531136@163.com (B.J.); cruhaichen@163.com (R.C.); 18846437313@163.com (Y.C.); zxm807646@163.com (X.Z.); 13555316428@163.com (Y.S.); 2 Guizhou Key Laboratory of Plateau Wetland Conservation and Restoration, Guizhou Normal University, Guiyang 550025, China; 3 Guizhou Engineering Laboratory for Quality Control & Evaluation Technology of Medicine, Guizhou Normal University, Guiyang 550025, China; 4 The Research Center for Quality Control of Natural Medicine, Guizhou Normal University, Guiyang 550025, China

**Keywords:** *Mori Fructus* polysaccharide (MFP-1), alcohol-associated liver disease (ALD), proteomics, metabolomics, lipidomics, multi-omics analysis

## Abstract

Alcohol-associated liver disease (ALD) is a prevalent chronic liver disease worldwide, with unclear pathogenesis and limited effective treatments. *Mori Fructus* polysaccharide (MFP-1) exhibits good therapeutic potential for ALD, but its mechanism remains unclear. This study aims to elucidate how MFP-1 mitigates ALD. An integrated multi-omics approach, encompassing quantitative proteomics, metabolomics, and lipidomics, was employed to systematically characterize the hepatic response to MFP-1 in ALD. MFP-1 coordinates metabolic reprogramming by regulating fatty acid synthesis and β-oxidation. It also enhances branched-chain amino acid catabolism via the 2-oxocarboxylic acid pathway, optimizing energy generation and amino acid utilization. MFP-1 protects against ALD by simultaneously targeting multiple metabolic vulnerabilities. These findings elucidate the mechanistic basis of MFP-1’s hepatoprotective effects and highlight its potential for improving ALD.

## 1. Introduction

Alcohol-associated liver disease (ALD) is a liver disorder characterized by impaired liver function due to excessive alcohol consumption [1]. ALD encompasses a spectrum of liver diseases, including fatty liver, hepatitis, liver fibrosis, cirrhosis, and hepatocellular carcinoma [2,3]. Its pathogenesis is driven by interconnected processes, including inflammation, oxidative stress, fibrosis, and metabolic disturbances. Globally, the incidence and mortality of ALD continue to rise, causing over 3 million deaths annually, with a five-year mortality rate exceeding 50% [4,5]. This poses a severe public health challenge. Notably, patients with ALD also face elevated risks of comorbid conditions such as hypertension and hypercholesterolemia [4].

Current clinical treatment strategies for ALD remain limited, and existing pharmacotherapies are often associated with significant adverse reactions. Consequently, there is an urgent need to develop efficient and safe alternative treatments. Natural bioactive compounds from medicinal and edible homologous plants present a promising avenue for discovery.

*Mori Fructus* (mulberry), a traditional medicinal food [6,7], is rich in various bioactive components [8]. Among these, *Mori Fructus* polysaccharides (MFP) are considered one of the key efficacy components. Research has shown that MFP possesses excellent antioxidant properties and can effectively reduce serum free fatty acid and triglyceride levels in diabetic mice [9]. Critically, MFP confers protection against palmitic acid-induced hepatocyte lipotoxicity by activating the Nrf2/ARE antioxidant signaling pathway [8]. Our previous works have identified MFP-1, a core polysaccharide fraction of MFP, which exhibits significant protective effects in ALD, alleviating both liver injury and lipid accumulation [10,11,12]. The potent antioxidant activity of MFP-1 is central to its hepatoprotective mechanism, directly countering the oxidative stress that is a hallmark of ALD progression.

However, the precise molecular targets, key signaling pathways, and the systems-level integration of MFP-1’s effects on protein, lipid, and metabolite networks remain incompletely elucidated.

The rapid advancement of systems biology has catalyzed transformative shifts in research paradigms. The multi-omics technologies, including proteomics, metabolomics, and lipidomics, constitute a robust research framework. This integrated approach not only enables comprehensive profiling of dynamic physiological states to identify novel drug targets and biomarkers, but also synergistically deciphers the complex interaction networks of natural products, thereby providing critical mechanistic insights into their multi-target therapeutic actions [13,14,15,16,17]. Proteomics involves the large-scale analysis of protein expression levels, post-translational modifications, and protein-protein interactions, providing deep insights into disease mechanisms such as cellular metabolism and other processes at the protein level. Metabolomics quantifies metabolites within an organism and examines their relative relationships to physiological and pathological changes. Lipidomics is the systematic analysis and identification of lipids and the molecules interacting with them in organisms, tissues, or cells. Its primary objective is to elucidate lipid structure and function, as well as to reveal the relationship between lipid metabolism and physiological and pathological processes of cells, organs, and the organism. While multi-omics technologies have been applied to study other polysaccharides, such as kelp polysaccharides in diabetes [18] or *Trichosporon* polysaccharide in oncology [19]. However, an integrated multi-omics analysis of MFP-1 in the context of ALD has not been reported.

Therefore, this study aims to systematically unveil the multi-target mechanism of MFP-1 intervention in ALD through integrated proteomics, metabolomics, and lipidomics analyses. This research will focus on elucidating the regulatory role of MFP-1 in core pathological processes of ALD, with particular emphasis on its antioxidant actions against hepatic oxidative stress, as well as its effects on inflammation and lipid metabolic disorders. This work seeks to clarify the molecular mechanisms of MFP-1 comprehensively and provide a solid theoretical and experimental basis for developing MFP-1 as a natural product-based therapeutic strategy for metabolic regulation in ALD.

## 2. Materials and Methods

### 2.1. Materials

Dithiothreitol (DTT), Iodoacetamide (IAA), 2-[4-(2-hydroxyethyl)piperazin-1-yl] ethanesulfonic acid (HEPES), Sodium deoxycholate (SDC), Ammonium bicarbonate (NH_4_HCO_3_), Hydroxylamine, Formic acid (FA), Protease inhibitor cocktail, and TFA were obtained from Sigma-Aldrich (St. Louis, MO, USA). Acetonitrile (ACE), H_2_O, and Acetone were purchased from ANPEL Laboratory Technologies (Shanghai) Inc. (Shanghai, China). Trypsin (sequence grade) purchased from Promega (Madison, WI, USA). Nonidet P40 (NP-40), NaCl, and SDS were purchased from Sangon Biotech (Shanghai, China). TMT Isobaric Label Reagent Set (6-plex, 10-plex, 16-plex) purchased from Thermo Scientific (Rockford, IL, USA). Enhanced BCA Protein Assay Kit purchase from Beyotime (Shanghai, China). Methanol, acetonitrile, isopropanol, and xylene were purchased from Chongqing Guangdong Chemical Co., Ltd. (Chongqing, China). Red Star Erguotou (56% vol.) from Beijing Red Star Co., Ltd. (Beijing, China). Bifendate dropping pills were produced by Wanbangde Pharmaceutical Group Co., Ltd. (Taizhou, China). Physiological salt water was purchased from Guangzhou Cologne Pharmaceuticals Co., Ltd. (Guangzhou, China). Methyl tert-butyl ether was purchased from Guiyang Ron Chemical Reagent (Guiyang, China). Water-free ethanol was purchased from Tianjin Zhiyuan Chemical Reagent Co., Ltd. (Tianjin, China). Alanine Aminotransferase (ALT) kits, aspartate aminotransferase (AST) enzyme activity kits, superoxide dismutase (SOD) assay kits, glutathione peroxidase (GSH-Px) activity kits, malondialdehyde (MDA) detection kits, and catalase (CAT) activity kits were purchased from Nanjing Jiancheng Bioengineering Institute Co., Ltd. (Nanjing, China). The acetyl-CoA carboxylase alpha (ACACA), and Fructose Biphosphate Aldolase B (ALDOB) ELISA kits were procured from Shanghai Jianglai Biotechnology Co., Ltd. (Shanghai, China). Except for acetonitrile of LC-MS grade, all other reagents are analytical grade and used directly after purchase.

MFP-1 was isolated and purified using the research methods of our team, and its structure as well as its anti-ALD activity have also been confirmed [10,12,20].

### 2.2. Animals and Model Preparation

Male SD rats (250~300 g) were used in this study. Animals provided by Changsha Tianqin Biotechnology Co., Ltd. (Changsha, China, license number: SCXK (X) 2022-0011). All rats were placed in a specific room (12 h alternating day and night, temperature 25 ± 2 °C, humidity 50%~70%) to provide a standard laboratory diet and water for rats and were cared for and treated according to the requirements of the Research Ethics Committee of Guizhou Normal University (Approval No. 2025050001). The experiment was conducted after a 7-day acclimation period.

Twenty-four rats were randomly assigned to four distinct groups, each containing six rats: control group (CN, administered normal saline); model group (ALD, administered an aqueous solution containing 56% vol ethanol (diluted from Red Star Erguotou baijiu) at 10 mL/kg body weight); bifendate-positive drug control group (Bif, administered bifendate (10 mL/kg) and an aqueous solution containing 56% vol ethanol (diluted from Red Star Erguotou baijiu) at 10 mL/kg body weight); and MFP-1 group (MFP-1, administered MFP-1 (150 mg/kg) and an aqueous solution containing 56% vol ethanol (diluted from Red Star Erguotou baijiu) at 10 mL/kg body weight). The Bif group and MFP-1 group received their respective drugs based on daily body weight, while the CN group and ALD group received an equal volume of normal saline. Two hours later, except for the CN group, all other groups were given 56% vol ethanol (10 mL/kg body weight) once daily for seven consecutive days. No deaths or adverse reactions occurred during the experiment.

Bifendate was selected as the positive control due to its well-documented hepatoprotective effects. It has been shown to reduce liver enzyme levels (ALT, AST) and oxidative stress markers (MDA, SOD) in liver injury models, thereby serving as a benchmark for validating the efficacy of our intervention [21,22].

The selection of MFP-1 dosage and alcohol usage, as well as the method of administration, are based on clinical dosages, reference literature, and previous research work [10,11].

Twelve hours after drug administration on the 7th day of the experiment, mouse blood was collected by puncturing the eyeball following anesthesia. The collected blood was centrifuged at 3000 r·min^−1^ for 15 min under 4 °C conditions to separate the serum, which was then stored at −80 °C for subsequent use. Subsequently, rats were euthanized by cervical dislocation. Liver tissues were obtained and weighed, and data was recorded, with photographs taken for archiving. Liver samples were divided into aliquots, rapidly frozen in liquid nitrogen, and stored at −80 °C for further testing.

### 2.3. Biochemical Analysis

The liver samples were weighed precisely to 0.1 g, then homogenized with 9 volumes of normal saline in an ice-water bath. Following centrifugation at 2500 r·min^−1^ for 10 min, the supernatant was harvested, which was then stored at −80 °C for subsequent use.

ALT (U/L), AST (U/L), SOD (U/mL), GSH-Px (U/L), MDA (nmol/mL), and CAT (U/mL) levels in the blood and/or liver tissues were quantified using kits from the Nanjing Jiancheng Bioengineering Institute (Nanjing, China) following the manufacturer’s instructions. The experiment was set up with 6 biological replicates (*n* = 6).

### 2.4. Proteomic Analysis

#### 2.4.1. Tissue Sample Treatment

Liver tissues were transferred into an EP tube, 300 μL of RIPA lysis buffer was added, and steel balls were introduced for ultrasonic-assisted homogenization at 35 Hz for 4 min. Following ultrasonication, the mixture was centrifuged at 12,000 r·min^−1^ for 10 min at 4 °C, and the resultant supernatant was transferred to a fresh EP tube. The extracted supernatant’s protein concentration was assessed using the BCA protein detection kit. The experiment was set up with 6 biological replicates (*n* = 6).

100 μg of total protein was diluted in pure water to achieve a final concentration of 1 mg/mL, followed by the addition of five times the volume of pre-cooled acetone. The mixture was then precipitated at −20 °C for a minimum of 16 h and centrifuged at 12,000 r·min^−1^ at 4 °C for 10 min. The supernatant was decanted, and the pellet was washed with 80% ice-cold acetone solution, followed by centrifugation to remove the supernatant. Protein precipitation, treated with DTT to disrupt disulfide bonds, was followed by the addition of IAA to alkylate the reduced disulfides. Post-proteolytic processing included TMT labeling, with equal volumes of labeled samples from each group mixed, after which TFA was introduced to precipitate SDC completely, thereby obtaining labeled peptide fractions. Peptide samples were subjected to overnight freeze-drying. Following redissolution in mobile phase A, analysis was conducted using an XBridge BEH C18 XPColumn (Waters Corporation, Milford, MA, USA). The flow rate was set at 0.3 mL/min. Mobile phase A consisted of a 10 mM ammonium acetate aqueous solution with pH 10, while mobile phase B was a 10 mM ammonium acetate, 10% H_2_O, 90% ACN solution also at pH 10. The gradient elution was as follows: mobile phase B from 5% to 30% in 40 min, then to 90% in 4 min, and held at 90% for 2 min before returning to 2% in 2 min to cycle. The collected fractions were combined into 12 components, vacuum-dried, and then frozen at −80 °C prior to nanoLC-MS/MS analysis.

#### 2.4.2. NanoLC-MS/MS Testing Conditions

2 μL of total peptides were separated using a nano-UPLC system (EASY-nLC1200) (Thermo Fisher Scientific, Waltham, MA, USA) and the resulting data were acquired with a mass spectrometer fitted with a nano-ion source (QExactive HFX) (Thermo Fisher Scientific, Waltham, MA, USA). Chromatographic separation was performed on a 100 μID × 15 cm reversed-phase chromatographic column (Reprosil-Pur120 C18-AQ, 1.9 μ, Dr. Maisch) (Dr. Maisch GmbH, Ammerbuch-Entringen, Germany) at a flow rate of 0.3 μL/min. The mobile phase was an acetonitrile-water-formic acid system. The mobile phase A was 0.1% formic acid-98% aqueous solution (acetonitrile was 2%), and the mobile phase B was 0.1% formic acid-80% acetonitrile solution (water was 20%). Gradient time according to the proportion of mobile phase B: 2–5% for 2 min, 5–22% for 68 min, 22–45% for 16 min, 45–95% for 2 min, and 95% for 2 min. Mass spectrometry analysis was performed using data-dependent acquisition (DDA) mode (positive ion detection mode). Full scan range 350–1600 *m*/*z*, resolution 120 k (200 *m*/*z*), AGC 3E6, maximum ion implantation time (maxIT) 30 ms; the 20 ions (top 20) with the highest intensity in the first-order scan were screened by quadrupole and subjected to fragment ion scanning after HCD cleavage. The quadrupole isolation window is 0.7 *m*/*z*, the normalized collision energy (NCE) is 32%, the AGC is 1E5, and the maxIT is 96 ms. The fixed minimum *m*/*z* of the two-stage scan is 110 (fixed first mass), and the resolution is 45 k. According to the peak width of the chromatographic peak, the dynamic exclusion time was set to 45 s; single charge and >6 valence ions do not undergo secondary scanning.

#### 2.4.3. Proteomic Data Analysis

Raw MS files were processed through the SpectroMine software (version 4.2.230428.52329) and its integrated Pulsar search engine. The MS spectra lists were queried against the UniProt species-level FASTA databases corresponding to rat (uniprot_Rattus norvegicus_10116_2023_09.fasta), applying carbamidomethylation (C) as a fixed modification and oxidation (M) and acetylation (protein N-term) as variable modifications. Trypsin digestion was employed as the protease, with an allowance for up to 2 missed cleavage sites. The false discovery rate (FDR) was set to 0.01 for both protein-spectrum matches (PSMs) and peptides. Peptide identification was constrained by a maximum precursor mass deviation of 20 ppm and a fragment mass deviation of 20 ppm. Unique peptides and Razor peptides were selected for protein quantification, while median values were used for normalization. All other parameters remained at default settings. The screening criteria were set at *p* < 0.05 for Students’ *t*-test and log_2_(FC) ≤ −0.263 or log_2_(FC) ≥ 0.263.

### 2.5. Metabolomics Analysis

#### 2.5.1. Blood Sample Processing

500 μL of plasma was taken and added into 1.5 mL of a pre-cooled methanol-acetonitrile-water system (5:3:2, *v*/*v*/*v*), vortexed, placed at −20 °C for 1 h, and centrifuged at 14,000 r·min^−1^ for 10 min. The supernatant was dried with a nitrogen-blowing instrument and stored in a refrigerator at −80 °C. Before the analysis, 500 μL of acetonitrile-water (1:1) was added to redissolve, vortexed, and centrifuged at 14,000 r·min^−1^ for 10 min, and the supernatant was filtered through a 0.45 μm filter membrane. Integrate the quality control samples with the test samples prior to conducting the testing. This ensures a comprehensive and accurate assessment of the test samples while maintaining the integrity of the quality control process. The experiment was set up with 6 biological replicates (*n* = 6).

#### 2.5.2. Tissue Sample Treatment

200 mg of liver samples were added to 400 μL of pre-cooled ultrapure water. Then 3.6 mL of acetonitrile/methanol (8:2, *v*/*v*) solution was added. The mixture was vortexed thoroughly and homogenized on ice, followed by centrifugation at 14,000 r·min^−1^ for 20 min at 4 °C. After centrifugation, the supernatant was collected to precipitate proteins, transferred to a new centrifuge tube, and dried under a stream of nitrogen (N_2_). The residue was stored at −80 °C until analysis.

Prior to analysis, the dried extract was reconstituted with acetonitrile-methanol (8:2, *v*/*v*)/ultrapure water (1:1, *v*/*v*), vortexed, and centrifuged at 14,000 r·min^−1^ for 20 min at 4 °C. The resulting supernatant was filtered through a 0.45 μm membrane filter and transferred to an autosampler vial.

Quality control (QC) samples were pooled from all test samples and analyzed alongside the batch. QC and test samples were injected in a randomized order to ensure data reliability and minimize instrument drift. The experiment included 6 biological replicates (*n* = 6).

#### 2.5.3. Chromatographic and Mass Spectrometric Conditions

The Waters ACQUITY UPLC HSS T3 (Waters Corporation, Milford, MA, USA) (100 mm × 2.1 mm, 1.8 μm) chromatographic column was equipped with the Dionex™ UliMate™ 3000 ultra-high-performance liquid chromatography separation system (Thermo Fisher Scientific, Waltham, MA, USA). The column temperature was 40 °C, the injection volume was 4 μL, the mobile phase A was acetonitrile (containing 0.1% formic acid), and the mobile phase B was water (containing 0.1% formic acid). The elution was performed at a flow rate of 0.3 mL/min. The gradient time was maintained at the ratio of mobile phase B: 95% for 1 min, 95–1% for 19 min, and 1% for 4 min.

The electrospray ionization (ESI) source was operated in positive and negative ion modes. Ion source gas 1 flow rate: 55 psi; ion source gas 2 flow rate: 55 psi; curtain gas flow rate: 35 psi; ion source temperature: 600 °C; ion source voltage: +5500 V to −4500 V; mother ion scanning range: 50~1000 Da; ion scanning range: 25~1000 Da; the accumulation time of parent ion scanning was 0.20 s, and the accumulation time of daughter ion scanning was 0.15 s. The secondary mass spectrometry used information-dependent acquisition (set to exclude isotopes within 4 Da, scanning 6 candidate ions per cycle), high sensitivity mode, decluster voltage of +60 V, and collision energy (35 + 15) eV.

#### 2.5.4. Metabolomics Data Analysis

The original data from blood samples and tissues were processed, and deviation values were filtered. Single peaks were removed to eliminate noise. Deviation values were filtered based on the relative standard deviation. Missing values were filtered, single peaks were excluded, and only peak area data with no more than 50% null values in any single group or across all groups were retained. The missing values in the original data are simulated, and the numerical simulation method is to fill the data standardization with the minimum value of one half. The total ion flow of each sample is normalized and then searched and sorted in the database. Finally, the LIMS 2 cloud platform was used for multivariate statistical analysis of the data.

### 2.6. Lipidomics Analysis

#### 2.6.1. Tissue Sample Treatment

A 0.1 g sample of liver tissue was placed in a centrifuge tube. Subsequently, 600 μL of pre-chilled ultrapure water was added and allowed to react for 1 min. This was followed by the addition of 2.4 mL of pre-chilled methyl tertiary-butyl ether, after which 720 μL of pre-chilled methanol was introduced. The mixed samples were sonicated for 20 min in low-temperature water and 30 min at room temperature and centrifuged at 14,000 r·min^−1^ for 15 min at 10 °C. The upper organic items were dried by a nitrogen-blowing instrument and stored in a refrigerator at −80 °C. Before the analysis, 600 μL of isopropanol/acetonitrile/water (65:30:5) solution was added to redissolve and centrifuged at 14,000 r·min^−1^ for 15 min at 10 °C. The samples to be tested were mixed as quality control (QC) samples. The experiment was set up with 6 biological replicates (*n* = 6).

#### 2.6.2. Chromatographic and Mass Spectrometric Conditions

The Waters ACQUITY UPLC HSS T3 (100 mm × 2.1 mm, 1.8 μm) column (Waters Corporation, Milford, MA, USA) was coupled with the Dionex^TM^ UliMate^TM^ 3000 ultra-high-performance liquid chromatography (UHPLC) system (Thermo Fisher Scientific, Waltham, MA, USA). The column temperature was 45 °C, the flow rate was 300 μL·min^−1^, mobile phase A was acetonitrile/aqueous solution (6:4), mobile phase B was acetonitrile/isopropanol solution (1:9), and the gradient time was according to the proportion of mobile phase B: 30% to maintain for 2 min, 100% to maintain for 23 min, and 30% to maintain for 10 min.

ESI-MS analysis was conducted in positive and negative ion modes. The operating conditions were as follows: heating temperature, 300 °C; sheath gas flow rate, 45 arb; auxiliary gas flow rate, 15 arb; purge gas flow rate, 1 arb; spray voltage, +3000 V to −2500 V; ion source temperature, 350 °C. The mass spectrometry scanning encompassed a range of 200–1800 Da for positive ions and 250–1800 Da for negative ions, with high-energy collision dissociation employed. Post-scanning, ten fragments were acquired.

#### 2.6.3. Lipidomics Data Analysis

The original data were preprocessed by Shanghai Baiqu Company (Shanghai, China), and the data were normalized. The response values of each lipid metabolite in QC samples were calculated, and the lipid metabolites with response values greater than 50% were removed. After the missing values were processed, the above data were standardized and converted, and the multivariate statistical analysis of the data was performed using the LIMS 2 cloud platform. The results of principal component analysis (PCA) and orthogonal partial least squares discriminant analysis (OPLS-DA) showed that there were differences in lipid metabolism among the groups. With VIP > 1, *p* < 0.05, and |log_2_(FC)| ≥ 0.263 as the screening conditions, the difference variables were obtained and analyzed.

### 2.7. Statistical Analysis

All quantitative data are reported as the mean ± standard error of the mean (mean ± SEM). Statistical significance between two groups was determined by an unpaired two-tailed Student’s *t*-test. Comparisons across multiple groups were performed using one-way ANOVA followed by an appropriate post hoc test. Data analysis and visualization were conducted with GraphPad Prism 9.0 (GraphPad Software, La Jolla, CA, USA), SPSS 26.0 (IBM, Armonk, NY, USA), R4.4.0 (R Foundation, Vienna, Austria), the Mai Microbial Informatics Cloud Platform 1.3.1, and standard microbiome bioinformatics pipelines. In all analyses, *p* < 0.05 was considered statistically significant.

## 3. Results

### 3.1. MFP-1 Ameliorates Biochemical and Oxidative Stress Markers in ALD

**Figure 1:**
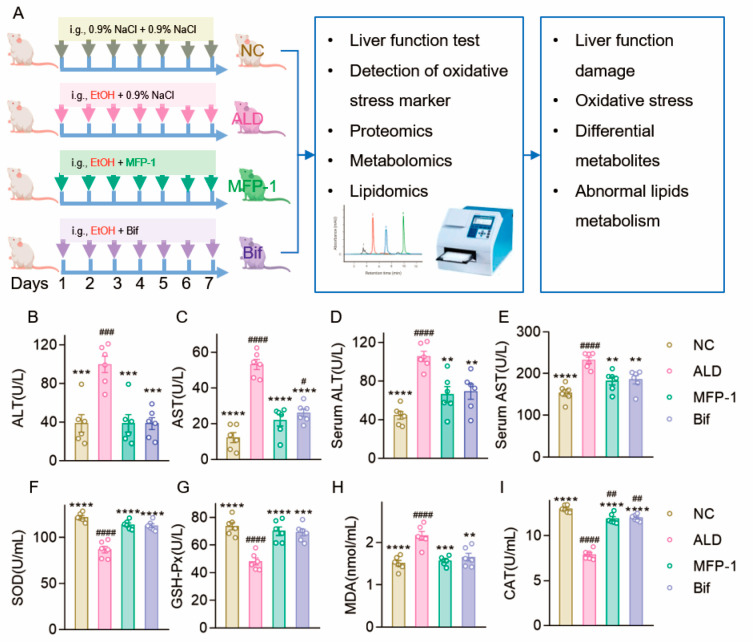
MFP-1 effectively mitigates ALD. (**A**) The schematic of experimental design. Arrows indicate gavage with 0.9% NaCl, alcohol, bifendate, or MFP-1 every day. i.g., represents intragastric administration; (**B**–**E**) The effect of MFP-1 on AST and ALT levels in liver tissue and serum; (**F**–**I**) The content or activity of SOD, GSH-Px, MDA, and CAT in the liver tissue. NC for the control group, ALD for the alcohol exposure treatment group, and MFP-1 represents the MFP-1 intervention group. The data are expressed as the mean ± SEM. *n* = 6. Compared with the ALD group, ** *p* < 0.01, *** *p* < 0.001, **** *p* < 0.0001; compared with the NC group, ^#^ *p* < 0.05, ^##^ *p* < 0.01, ^###^ *p* < 0.001, ^####^ *p* < 0.0001.

To evaluate the therapeutic potential of MFP-1 against ALD, we established a rat model of ALD and administered MFP-1 as an intervention (Figure 1A). Serum and liver levels of ALT and AST, key biochemical markers of hepatic injury, were significantly elevated in the ALD group compared to the normal control (NC) group (*p* < 0.0001; Figure 1B–E), confirming successful model induction. MFP-1 markedly reduced these enzyme levels by 61.37% (ALT) and 58.95% (AST) in liver tissue compared to the ALD group (*p* < 0.001), demonstrating substantial hepatoprotection. Notably, the MFP-1 group’s ALT and AST levels were statistically comparable to those in both the bifendate-positive control (Bif) and NC groups *(p* > 0.05). This suggests that MFP-1 effectively mitigates ALD.

We next assessed the status of oxidative stress, a core driver of ALD pathology, by measuring SOD, GSH-Px, MDA, and CAT (Figure 1F–I). The ALD group exhibited a pronounced oxidative stress state, characterized by significantly decreased activities of the antioxidant enzymes SOD, GSH-Px, and CAT (*p* < 0.0001) and a significant increase in the lipid peroxidation product MDA (*p* < 0.0001) relative to the NC group. Both MFP-1 and bifendate treatments effectively counteracted these changes, significantly elevating antioxidant enzyme activities and reducing MDA content, thereby indicating a potent capacity to alleviate alcohol-induced oxidative stress.

### 3.2. Proteomic Alterations in the Liver Induced by MFP-1 Intervention

To elucidate the protein-level changes associated with MFP-1 intervention, a comparative proteomic analysis was conducted on liver tissues from the ALD and MFP-1 groups using TMT-based quantitative proteomics (Table S1). This approach provided a high-resolution profile of protein abundance variations.

**Figure 2:**
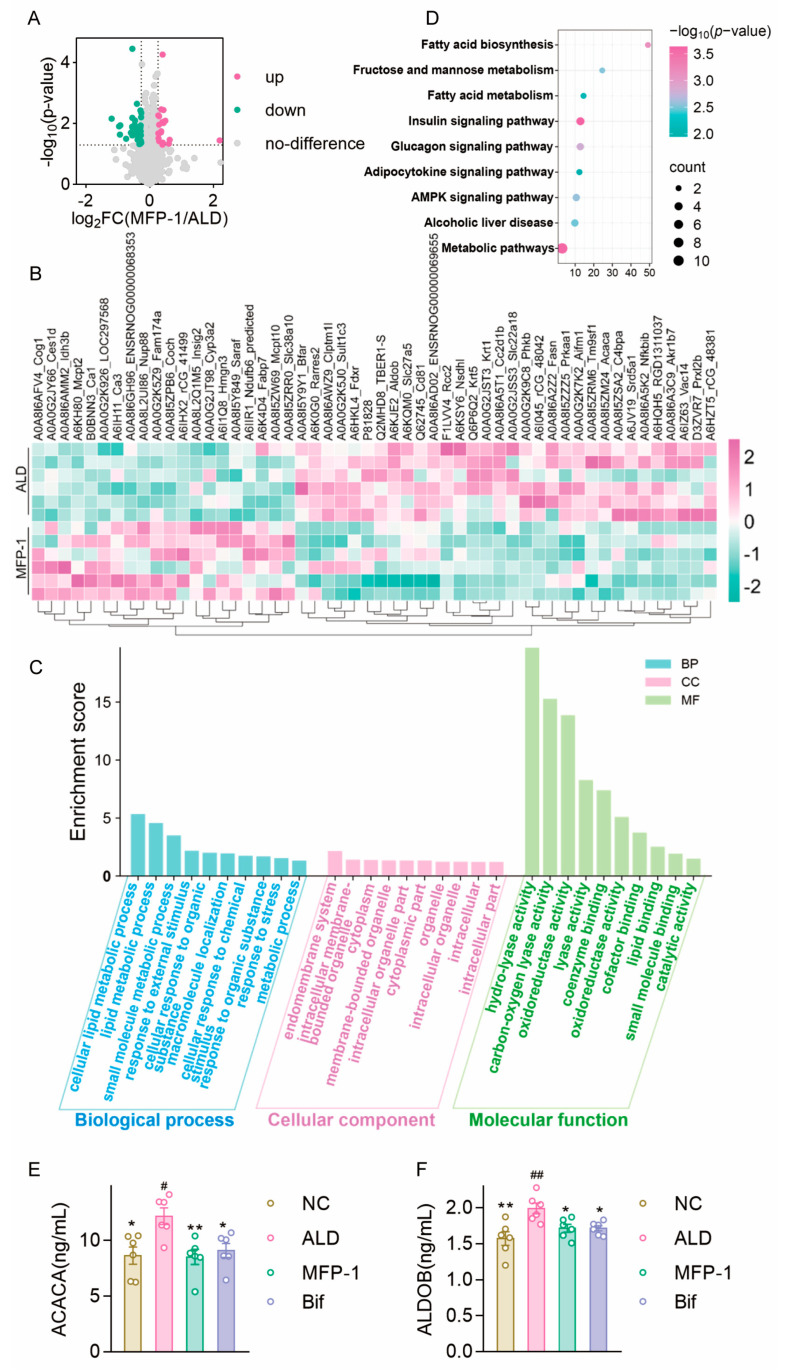
Proteomic analysis of differentially expressed proteins in MFP-1 group compared with the ALD group. (**A**) Volcano diagram; (**B**) Heatmap showing enrichment of differentially expressed proteins between MFP-1 and ALD groups; (**C**) GO enrichment analysis of differentially expressed proteins between MFP-1 and ALD groups; (**D**) KEGG pathway analysis on liver differential proteins. *n* = 6. (**E**) The content of ACACA. *n* = 6. Compared with the ALD group, * *p* < 0.05, ** *p* < 0.01; compared with the NC group, ^#^ *p* < 0.05. (**F**) The content of ALDOB. *n* = 6. Compared with the ALD group, * *p* < 0.05, ** *p* < 0.01; compared with the NC group, ^##^ *p* < 0.01.

Comparative analysis between the ALD and MFP-1 groups identified 55 differentially expressed proteins (DEPs) (*p* < 0.05, fold change (FC) >1.2 or <0.263). Among these, 20 proteins were upregulated and 35 proteins were downregulated in the MFP-1 group relative to the ALD group (Figure 2A, Table 1), highlighting the specific impact of MFP-1 on the hepatic proteome.

A clustering heatmap of these DEPs revealed distinct expression patterns, clearly separating the MFP-1 group (green) and the ALD group (pink) into two major clusters (Figure 2B). Intra-group samples clustered tightly, indicating consistent biological profiles within each condition. Key proteins significantly enriched in the ALD group included carboxylesterase 1D (Ces1d), ENSNOGO000008353, and coenzyme Q biosynthesis protein 1 (Coq1), with z-scores exceeding 2.5, indicating statistical significance after multiple testing corrections. In contrast, proteins such as carbonic anhydrase 3 (Ca3) and Vec14 were predominantly expressed in the MFP-1 group.

Gene Ontology (GO) enrichment analysis of the DEPs provided functional insights into the biological impact of MFP-1 (Figure 2C). Cellular compartment (CC) analysis revealed that proteins regulated by MFP-1 are primarily found in the endomembrane system, intracellular membrane-bound organelles such as mitochondria and lysosomes, cytoplasm, endoplasmic reticulum, Golgi apparatus, other cellular compartments, and extracellular matrix. Biological process (BP) analysis (Figure 2C) indicates that the DEPs are mainly involved in cellular lipid metabolism (including both fatty acid oxidation and synthesis), lipid metabolism pathways (such as triglyceride and phospholipid production), and small molecule metabolism (encompassing carbohydrate and amino acid metabolism). This pattern suggests a broad influence of MFP-1 on core metabolic pathways disrupted in ALD.

Molecular function (MF) analysis (Figure 2C) demonstrated that the majority of the DEPs displayed a diverse range of enzymatic and binding activities, underscoring their roles in catalyzing reactions and mediating molecular interactions. Notably, there was significant enrichment in oxidoreductase activity and antioxidant activity, functions directly pertinent to countering oxidative stress. Other prominent functional categories included hydrolase activity, carbon-oxygen lyase activity, and various binding activities such as coenzyme binding and lipid binding. The enrichment of oxidoreductase and antioxidant functions at the molecular level provides a direct proteomic correlate to the observed biochemical amelioration of oxidative stress by MFP-1 treatment.

KEGG pathway enrichment analysis further contextualized the DEPs within specific biological processes (Figure 2D–F). The most significantly altered pathways were fatty acid biosynthesis, fatty acid metabolism, the AMPK signaling pathway, and the insulin signaling pathway. Key DEPs within these pathways, such as Acaca and fatty acid synthase (Fasn) in fatty acid biosynthesis, are central regulators of lipid homeostasis. Their modulation by MFP-1 suggests a targeted intervention into the dysregulated lipid metabolism that characterizes ALD. The involvement of the AMPK and insulin signaling pathways further implies that MFP-1 may ameliorate ALD-associated metabolic disturbances, including insulin resistance, by influencing these critical energy-sensing and hormonal regulatory networks.

In summary, the proteomic profile indicates that MFP-1 intervention induces a coordinated shift in the hepatic proteome. This shift is characterized by an enhanced antioxidant capacity (via oxidoreductase enrichment) and a concerted modulation of central metabolic pathways governing lipid and energy homeostasis. These proteomic changes align with the observed phenotypic improvement and provide a molecular framework for understanding MFP-1’s hepatoprotective mechanism.

**Table 1:** The top 20 differentially expressed proteins between the ALD and MFP-1 treatment groups (*n* = 6).

Number	Gene Name	Accession	Number	Gene Name	Accession
1	rCG_41499	A6IHX2	11	Ca3	A6IH11
2	Mcpt2	A6KH80	12	Hmgn3	A6I1Q8
3	Cyp3a2	A0A0G2JT98	13	Coch	A0A8I5ZPB6
4	RGD1311037	A6HQH5	14	Acaca	A0A8I5ZM24
5	Srd5a1	A6JV19	15		P81828
6	Rarres2	A6K0G0	16	Bfar	A0A8I5Y9Y1
7	Krt5	Q6P6Q2	17	Clptm1l	A0A8I6AWZ9
8	Akr1b7	A0A8I6A3C9	18	Nsdhl	A6KSY6
9	Krt1	A0A0G2JST3	19	ENSRNOG00000069655	A0A8I6AD02
10	Phkb	A0A0G2K9C8	20	Cc2d1b	A0A8I6A5T1

### 3.3. MFP-1 Modulates Serum Metabolic Alterations Profile in ALD

**Figure 3:**
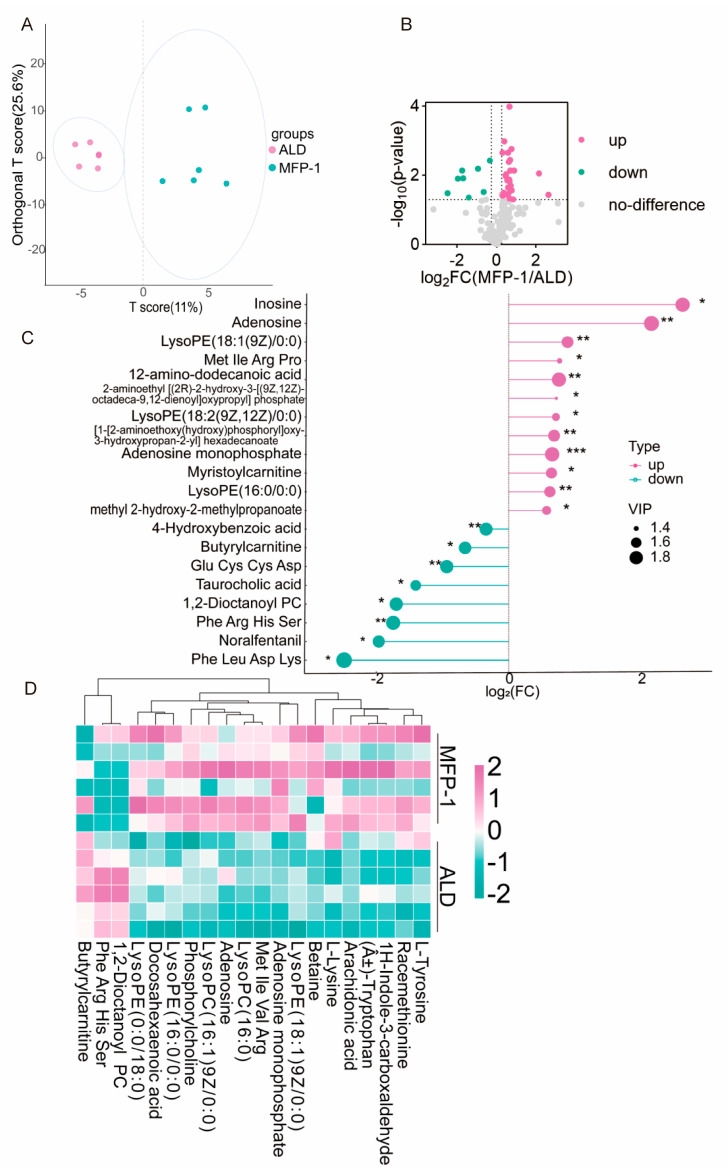
Metabolomics analysis of differential metabolites in blood samples between the MFP-1 group and the ALD group. (**A**) OPLS-DA analysis map of the MFP-1 group and ALD group; (**B**) volcano map of the ALD group and MFP-1 group; (**C**) matchstick map of the ALD group and MFP-1 group. VIP (variable importance in projection) scores indicate the contribution of metabolites to group separation. * *p* < 0.05, ** *p* < 0.01, *** *p* < 0.001; (**D**) Hierarchical clustering heat map of the ALD group and MFP-1 group (green down, pink up). *n* = 6.

To elucidate the metabolic alterations induced by ALD and the effects of MFP-1 intervention, we performed a comprehensive serum metabolomics analysis (Tables S2 and S3). OPLS-DA was performed to identify the key metabolic features between the ALD and MFP-1 groups. The model was rigorously validated through 200 permutation tests (*p* < 0.01), with goodness-of-fit (R^2^Y = 0.55) and predictive ability (Q^2^ = 0.57) assessed to guard against overfitting. Overfitting was assessed by comparing R^2^Y and Q^2^ values; models with Q^2^ < 0.5 were excluded. The predictive component (T score) explained 11.0% of variance, while the orthogonal component (T score) explained 25.6% of variance, indicating distinct metabolic phenotypes between ALD and MFP-1 groups (Figure 3A).

Differential metabolite screening with a Student’s *t*-test (*p* < 0.05) and a fold change threshold (|log_2_FC| ≥ 0.263) identified 30 significantly altered metabolites (Figure 3B and Table 2). Among these, 22 were upregulated and 8 were downregulated in the MFP-1 group compared to the ALD group. This asymmetric shift suggests a global metabolic reprogramming favoring anabolic processes. Upregulated metabolites were enriched biosynthetic intermediates and included amino acid precursors (glutamate and alanine), nucleotide building blocks (ribose-5-phosphate and uridine diphosphate), and lipid synthesis intermediates (acetyl-CoA and fatty acyl-CoA). Notably, adenosine monophosphate (AMP) was significantly upregulated (*p* = 0.0028, log_2_FC = 0.6), suggesting alterations in purine metabolism and cellular energy sensing. Conversely, downregulated metabolites were dominated by catabolic products (butyrate and acetate) and oxidative stress byproducts (MDA and 4-hydroxynonenal (4-HNE)). The downregulation of β-hydroxybutyrate (a ketone body) and short-chain fatty acids (SCFAs) was observed.

A comprehensive analysis of differentially enriched metabolites identified significant metabolic profile differences between the MFP-1 group and the ALD group (Figure 3C). The results revealed that upregulated metabolites were enriched in glycolysis, fatty acid biosynthesis, and aminoacyl-tRNA biosynthesis, while downregulated metabolites were associated with fatty acid degradation and oxidative phosphorylation. Adenosine monophosphate (AMP) was significantly upregulated (*p* = 0.002840, log_2_FC = 0.6), indicating abnormalities in purine metabolism and cellular energy homeostasis. Concurrently, the synergistic elevation of multiple metabolite classes—including phosphocholine (2.3-fold increase), lysophosphatidylcholine (16:0), amino acids and derivatives (methionine, isoleucine, valine, and arginine), and racemic methionine (1.8-fold increase)—suggests that MFP-1 may promote a metabolic state characterized by enhanced anabolic activity. Hierarchical clustering analysis (Figure 3D) further confirmed two distinct metabolic clusters. The ALD group was characterized by elevated Met Ile Val Arg, LysoPC(16:0), and myristoylcarnitine, alongside elevated phospholipid compounds such as PC(O-18:1(10E)/2:0). These findings reflect accelerated membrane turnover, activated inflammatory processes, and enhanced catabolic states of lipid metabolism and amino acid utilization. In contrast, the MFP-1 group showed accumulation of antioxidant substances (such as 4-hydroxybenzoic acid, taurine, and norepinephrine) and phosphocholine. This metabolic signature supports the premise that MFP-1 alleviates ALD, in part, by counteracting oxidative stress and promoting a systemic metabolic shift toward homeostasis.

**Table 2:** The top 20 differentially expressed serum metabolites between the ALD and MFP-1 treatment groups (*n* = 6).

Number	Name	Number	Name
1	Inosine	11	[1-[2-aminoethoxy(hydroxy)phosphoryl] oxy-3-hydroxypropan-2-yl] hexadecanoate
2	Adenosine	12	1H-Indole-3-carboxaldehyde
3	LysoPE(18:1(9Z)/0:0)	13	Adenosine monophosphate
4	Asn Pro Trp His	14	Docosahexaenoic acid
5	Met Ile Arg Pro	15	Butyrylcarnitine
6	12-amino-dodecanoic acid	16	Glu Cys Cys Asp
7	2-aminoethyl [(2R)-2-hydroxy-3-[(9Z,12Z)-octadeca-9,12-dienoyl]oxypropyl] phosphate	17	Taurocholic acid 1,2-Dioctanoyl PC
8	LysoPE(18:2(9Z,12Z)/0:0)	18	Phe Arg His Ser
9	Arachidonic acid	19	Noralfentanil
10	Inosine	20	Phe Leu Asp Lys

### 3.4. MFP-1 Improves Liver Metabolic Alterations Caused by ALD

**Figure 4:**
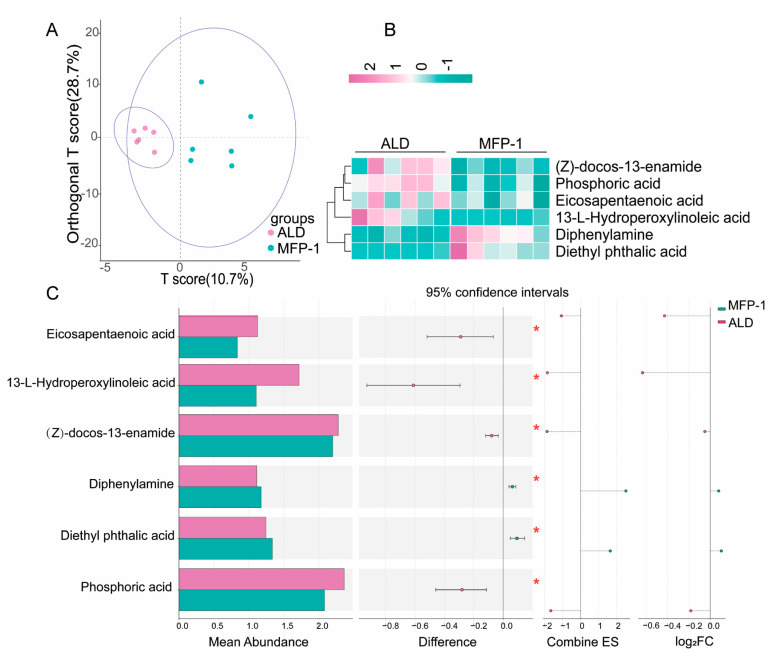
Metabolomics analysis of differential metabolites in liver tissues between the MFP-1 group and the ALD group. (**A**) OPLS-DA analysis map of the MFP-1 group and ALD group; (**B**) Hierarchical clustering heat map of ALD group and MFP-1 group (green down, pink up); (**C**) Abundance profile of major differential metabolites of ALD group and MFP-1 group,* *p* < 0.05. *n* = 6.

The OPLS-DA score plot revealed clear separation between ALD (pink) and MFP-1 (cyan) groups (Figure 4A, Tables S4 and S5). The model demonstrated high goodness-of-fit (R^2^Y = 0.82) and predictive ability (Q^2^ = 0.98), with permutation testing (*p* < 0.01) confirming robustness. The predictive component (T score) explained 10.7% of variance, while the orthogonal component (T score) explained 28.7% of variance, indicating distinct metabolic phenotypes. The distinct clustering of ALD and MFP-1 groups indicates MFP-1 can alter the metabolic profile of ALD. The results of this study are consistent with relevant research that links metabolic dysregulation to liver disease pathology.

Metabolite abundance profile analysis (Figure 4B) was performed on the metabolomics data of the MFP-1 and ALD groups. The pronounced divergence in metabolite abundance suggests group-specific metabolic adaptations. ALD samples showed elevated levels of pro-inflammatory lipid peroxidation products like 13-L-Hydroperoxylinoleic acid. MFP-1 samples exhibited an increased abundance of Diethyl phthalic acid, a potential marker of enhanced xenobiotic metabolism, indicating altered membrane lipid metabolism.

Statistical analysis of metabolomic data revealed significant differences between the ALD and MFP-1 groups (*p* < 0.05). Diphenylamine (log_2_FC = 0.48, *p* = 0.016), 13-L-hydroperoxylinoleic acid (log_2_FC = 0.42, *p* = 0.024), and Eicosapentaenoic acid (EPA) (log_2_FC = 0.35, *p* = 0.032) were elevated in ALD, suggesting oxidative stress activation. Oxidative stress leads to lipid peroxidation, a process where free radicals attack polyunsaturated fatty acids in cell membranes, resulting in the formation of oxidized lipid metabolites (13-L-hydroperoxylinoleic acid). EPA, an omega-3 polyunsaturated fatty acid, is a substrate for oxidative stress-induced peroxidation. Elevation of EPA in ALD may reflect increased bioavailability resulting from metabolic perturbations or heightened oxidative challenge. In contrast, Z-docos-13-enamide (log_2_FC = −0.50, *p* = 0.020) and phosphoric acid (log_2_FC = −0.32, *p* = 0.015) were enriched in MFP-1 (Figure 4C). The upregulation of diphenylamine in ALD aligns with its role as a compound that can participate in or be a byproduct of lipid peroxidation reactions, further supporting the presence of oxidative stress in this group. Z-docos-13-enamide in MFP-1 may indicate altered membrane lipid metabolism. Phosphoric acid differences, with lower levels in ALD and higher in MFP-1, imply potential shifts in energy metabolism pathways; phosphoric acid is a key component in ATP synthesis and energy transfer reactions, and its altered abundance could reflect changes in glycolysis, oxidative phosphorylation, or other metabolic processes involved in energy production and utilization. This indicates that MFP-1 can improve oxidative stress, membrane lipid metabolism, and energy metabolism during the ALD process.

### 3.5. MFP-1 Ameliorates Lipid Metabolic Alterations in the Liver Caused by ALD

**Figure 5:**
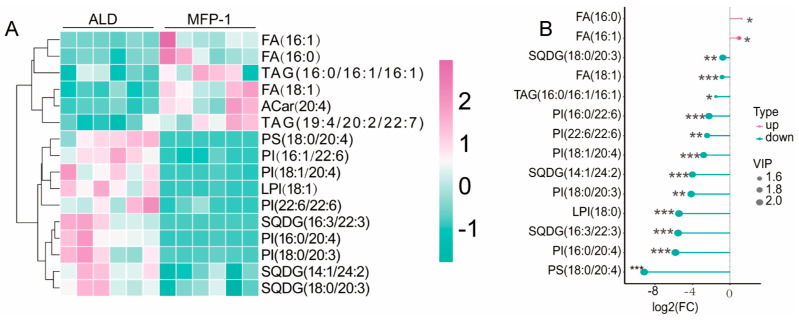
Lipidomics analysis of differential metabolic lipids in livers between the MFP-1 group and ALD group. (**A**) Hierarchical clustering heatmap of differential lipid biomarkers in the ALD and MFP-1 groups. pink is up-regulated, green is down-regulated; (**B**) matchstick map of the ALD group and MFP-1 group. VIP (Variable importance in projection) scores reflect the contribution of lipids to group separation. * *p* < 0.05, ** *p* < 0.01, *** *p* < 0.001 (*n* = 6 per group).

Figure 5A, Table 3 and Tables S6 and S7 revealed distinct metabolic profiles between the ALD and MFP-1 groups. Key lipid species such as FA(16:0) and FA(18:1) showed significantly higher abundance in the ALD group, whereas SODG(18:4/20:3) and PI(18:0/20:4) were enriched in the MFP-1 group. Cluster analysis indicated strong internal consistency within the ALD group, while the MFP-1 group exhibited greater heterogeneity. These findings suggest differential regulation of fatty acid metabolism and membrane lipid composition between the two conditions. The enrichment of saturated and monounsaturated fatty acids in the ALD group may reflect increased lipid storage or inflammatory responses, which is consistent with previous reports linking these lipids to metabolic stress. In contrast, the elevated levels of polyunsaturated fatty acid derivatives in MFP-1 indicate enhanced membrane fluidity or signaling activity and contribute to cell communication and immune regulation.

The differences in the abundance of differential metabolites between the MFP-1 group and the ALD group were analyzed using the matchstick plot analysis method (Figure 5B). A total of 14 lipid features were analyzed, showing significant differences between the MFP-1 and ALD groups. Most features exhibited higher mean abundance in the ALD group, particularly TAG(16:0/16:1/16), SQDG(16:3/22:3), and PI(22:6/22:6), with *p*-values as low as 0.007382 (**). These differences were further supported by negative log_2_FC values and large negative combined effect size (ES) values, indicating substantial enrichment in the ALD group. In contrast, FA(16:1) and FA(18:1) showed higher abundance in MFP-1, with positive log_2_FC and combined ES values. The results suggest distinct lipidomic profiles between the two groups, reflecting metabolic shifts. The enrichment of specific phospholipids and glycolipids in the ALD group suggests enhanced membrane dynamics or inflammatory responses, implying that MFP-1 can inhibit membrane fluidity and inflammatory responses during the ALD process.

**Table 3:** The top 15 differentially expressed metabolites of liver between the ALD and MFP-1 treatment groups. (*n* = 6).

Number	Name	Number	Name	Number	Name
1	FA(16:0)	6	PI(18:0/20:3)	11	SQDG(16:3/22:3)
2	FA(18:1)	7	PI(18:1/20:4)	12	SQDG(14:1/24:2)
3	FA(16:1)	8	PI(16:0/22:6)	13	PS(18:0/20:4)
4	LPI(18:0)	9	PI(22:6/22:6)	14	TAG(16:0/16:1/16:1)
5	PI(16:0/20:4)	10	SQDG(18:0/20:3)		

### 3.6. Comprehensive Analysis of Proteomics, Metabolomics, and Lipidomics

We integrated the results of metabolomics and lipidomics analyses. A total of 410 lipids and metabolites were detected, covering phospholipids, sphingolipids, glycerides, fatty acid derivatives, and various small-molecule metabolites (such as amino acids, organic acids, nucleotides, and carbohydrate metabolites). This provided a rich data foundation for a comprehensive analysis of ALD-related metabolic alterations.

A total of 57 lipids and metabolites with significantly differential expression were identified. Among them, 17 showed an upregulated trend and 40 showed a downregulated trend. These differential substances may serve as potential biomarkers or therapeutic targets.

**Figure 6:**
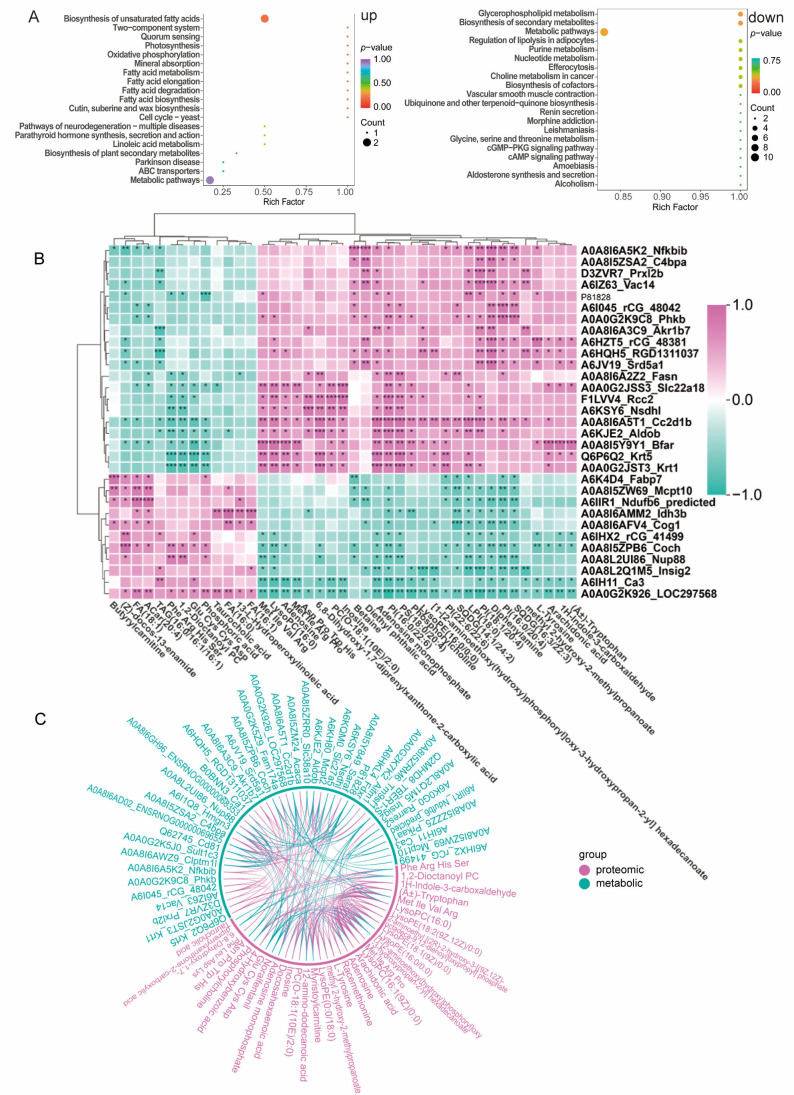
Comprehensive analysis of proteomics, metabolomics, and lipidomics between the MFP-1 group and ALD group. (**A**) KEGG enrichment analysis of metabolites and metabolic lipids in ALD and MFP-1 groups. Count the number of enriched metabolites; (**B**) Pearson correlation analysis between proteins, lipids, and metabolites in ALD and MFP-1 groups. Pink is a positive correlation, and green is a negative correlation. *n* = 6. * *p* < 0.05, ** *p* < 0.01, *** *p* < 0.001; (**C**) Multi-omics and chord diagrams analysis between proteins, lipids, and metabolites in ALD and MFP-1 groups.

The results of KEGG pathway enrichment analysis (Figure 6A) indicate that the main enrichment is in key biological processes such as metabolic pathways, unsaturated fatty acid biosynthesis, fatty acid metabolism, fatty acid elongation, fatty acid degradation, fatty acid cofactor biosynthesis, purine metabolism, and glycerophospholipid metabolism. This indicates that these pathways may play an important role in the occurrence and development of ALD, and their dysregulation may be closely related to lipid metabolic abnormalities, energy imbalance, and inflammatory responses.

To further understand the mechanism of MFP-1 in acute ALD in rats, we conducted a joint analysis of proteomics, metabolomics, and lipidomics. Correlation analysis was used to evaluate the potential correlation between proteins, lipids, and metabolites (Figure 6B,C). A total of 55 proteins that could distinguish between the ALD and MFP-1 groups were identified, including TBER1-S, Cc2d1b, Aldob, Bfar, Krt5, rCG_48042, Phkb, Acaca, Rarres2, Rcc2, Nsdh1, LOC297568, Insig2, Nup88, Hmgn3, Saraf, Cog1, and Ca3. Coch, and Fam174a. These proteins exhibited numerous correlations with lipid or metabolite molecules, such as fatty acid derivatives (palmitic acid and oleic acid), bile acids (taurocholic acid (TAC) and glycocholic acid), and amino acid metabolites (alanine, glutamate, and serine), suggesting a potential regulatory role in the metabolic disorder of liver tissue during acute ALD.

Specifically, proteins like Acaca, a key enzyme in fatty acid synthesis, showed strong positive correlations with elevated levels of fatty acid derivatives in the ALD group. This indicates that MFP-1 may inhibit Acaca activity to reduce de novo lipogenesis and subsequent lipid accumulation. Similarly, Insig2, which regulates cholesterol biosynthesis by controlling sterol regulatory element-binding protein (SREBP) processing, was found to be correlated with altered bile acid profiles, suggesting a role in modulating bile acid homeostasis and preventing cholestatic injury. Additionally, Aldob, which is involved in glycolysis and fructose metabolism, exhibited negative correlations with amino acid metabolites. This implies that MFP-1 may restore glycolytic flux and amino acid utilization, thereby mitigating oxidative stress and inflammatory pathways triggered by metabolic imbalance. These findings collectively highlight the multifaceted impact of MFP-1 on hepatic metabolism, linking protein expression changes to lipid and metabolite perturbations, and underscore its therapeutic potential in ameliorating ALD through the regulation of lipid accumulation, oxidative stress, and inflammatory responses.

## 4. Discussion

The liver functions as the primary organ responsible for alcohol catabolism and detoxification. Excessive alcohol consumption is the leading cause of liver disease [23]. Consequently, the identification of natural products for the prevention and management of ALD is critically important. Plant polysaccharides can mitigate various forms of liver injury. Among them, MFPs have anti-oxidation, hypoglycemic, anti-obesity, and other effects [24,25]. Our prior research indicates that MFP exhibits a protective effect against ALD [6,10,11]. However, the specific mechanism remained unclear.

This study used a rat ALD model to show that MFP-1 alleviates liver injury. Using biomarkers and pathological assessments, we found that MFP-1 intake protected against ALD. Subsequently, proteomics, metabolomics, lipidomics analysis, and integrated multi-omics analysis of rat serum or liver tissue were carried out to investigate the potential mechanism of MFP-1 in treating ALD.

Pathological analysis showed that MFP-1 intervention significantly alleviated liver tissue injury in ALD rats (*p* < 0.05). Proteomics, metabolomics, and lipidomics analyses were conducted on the MFP-1 group and ALD group. Key metabolites identified include 4-hydroxybenzoic acid, TAC, hypoxanthine, L-tyrosine, 3′-AMP, PI(16:0/22:6), PC(16:0/18:1), adenosine, phosphocholine, adenosine monophosphate, and glutathione. Differentially expressed proteins included Ca3, Cyp3a2, Idh3b, Acaca, Fasn, Slc27a5, Nsdhl, Aldob, Srd5a1, Prxl2b, and Akr1b7. These substances are mainly enriched in related metabolic pathways such as “Metabolic Pathways”, “Folate Biosynthesis”, “Amino Acid Biosynthesis”, and “2-Oxocarboxylic Acid Metabolisms”.

Liver diseases are linked to altered metabolic pathways. In this study, MFP-1 treatment significantly downregulated Acaca and Fasn, key enzymes in de novo lipogenesis. Acaca is a rate-limiting enzyme in fatty acid synthesis [26]. Fatty acid synthase is a multifunctional homodimeric enzyme protein serving as the primary enzyme for synthesizing and metabolizing dietary carbohydrates into fatty acids. Acaca and Fasn are validated targets for nonalcoholic fatty liver disease (NAFLD)/nonalcoholic steatohepatitis (NASH) therapy [27,28], with their inhibitors reducing hepatic lipid accumulation. Concurrently, MFP-1 altered phospholipid profiles (phosphatidylinositol PI(16:0/22:6) and phosphatidylcholine PC(16:0/18:1)), critical for membrane integrity and lipid transport. Within the metabolic pathway, several differential metabolites and proteins are implicated in lipid metabolism. Research has demonstrated that long-term intervention with TAC can inhibit the growth of HepG2 cells and induce apoptosis, which is beneficial for the treatment of patients with liver cancer [29]. Phosphorylcholine, an important component of the plasma membrane [30], was also modulated, aligning with its reported role in slowing NAFLD [31]. Carbonic anhydrase is a zinc-containing metalloenzyme. Studies have demonstrated that Squalene epoxidase and Ca3 can synergistically enhance NASH treatment in mice. These data suggest MFP-1 mitigates ALD by suppressing lipogenesis and restoring phospholipid homeostasis.

Differential metabolites (L-tyrosine and adenosine monophosphate) and proteins (Idh3b, Aldob, and Akr1b7) implicated MFP-1 in amino acid and energy metabolism. L-tyrosine is a crucial nutritional essential amino acid, critical for metabolism as well as growth and development in humans and animals [32]. L-tyrosine upregulated amino acid biosynthesis and 2-oxocarboxylic acid metabolism pathways, potentially reducing inflammation via microbial metabolism crosstalk [33]. Aldob, a glycolytic enzyme, was downregulated, which may alleviate insulin resistance by inhibiting PI3K-AKT pathway hyperactivation [34]. AKR1B7, an aldose reductase engaged in detoxification, is the principal enzyme catalyzing isohexaldehyde reduction [35,36]. AKR1B7 was suppressed, aligning with its role in reducing lipid peroxides [37]. Together, these changes suggest MFP-1 reprograms amino acid utilization and glycolytic flux to support hepatic energy homeostasis.

MFP-1 enhanced antioxidant capacity by increasing 4-hydroxybenzoic acid (a phenolic antioxidant) and modulating Prxl2b (a peroxiredoxin). 4-hydroxybenzoic acid, previously linked to cadmium-induced renal injury amelioration [38], likely scavenges reactive oxygen species (ROS), while Prxl2b reduces peroxides. These effects, coupled with folate biosynthesis pathway activation, support MFP-1’s role in mitigating oxidative stress—a key driver of ALD pathogenesis.

Key proteins were identified via omics correlations without independent validation. Future studies will validate targets and explore human-relevant models.

## 5. Conclusions

This study integrated proteomic, lipidomic, and metabolomic analyses to systematically elucidate the multi-target protective mechanisms of MFP-1 against ALD. The hepatoprotective effects operate through synergistic mechanisms: (1) Lipid regulation improvement. MFP-1 reduces hepatic lipid accumulation and lipotoxicity via dual regulation: enhancing fatty acid β-oxidation enzymes while suppressing fatty acid synthase through ACCα inhibition and Insig2 upregulation; and (2) Energetic & amino acid reprogramming. By activating rate-limiting enzymes in the 2-ketoglutarate pathway, MFP-1 promotes branched-chain amino acid oxidation and ATP generation. Concurrent Aldob activation restores glycolytic flux and amino acid utilization efficiency; (3) Enhanced antioxidant capacity. MFP-1 mitigates oxidative stress by scavenging reactive oxygen species and enhances detoxification capacity, providing a hepatoprotective barrier against alcohol metabolites; (4) Metabolic support. Modulation of folate cycle-driven one-carbon metabolism ensures essential amino acid biosynthesis and transport. Although MFP-1 demonstrated significant efficacy and favorable safety in animal models, limitations include sample size constraints and interspecies metabolic differences. Future work will validate targets and translate findings to human-relevant models. Collectively, this study not only elucidates the molecular mechanism by which MFP-1 exerts hepatoprotective effects through its actions on lipid metabolism, energy homeostasis, and redox balance but also provides a new theoretical basis and potential therapeutic targets for the prevention and treatment of metabolic liver diseases, demonstrating good translational medicine prospects.

## Data Availability

The original contributions presented in this study are included in the article and Supplementary Materials. Further inquiries can be directed to the corresponding author.

## Supplementary Materials

The following supporting information can be downloaded at: https://www.mdpi.com/article/10.3390/antiox15040443/s1, Table S1: Proteomics date; Table S2: Metabolomics data of serum under the negative ion mode; Table S3: Metabolomics data of serum under the postive ion mode; Table S4: Metabolomics data of liver under the negative ion mode; Table S5: Metabolomics data of liver under the postive ion mode; Table S6: Lipidomics data of liver under the negative ion mode; Table S7: Lipidomics data of liver under the postive ion mode.

## Abbreviations

The following abbreviations are used in this manuscript:

ALD	Alcohol-associated liver disease
MFP-1	*Mori Fructus* polysaccharides-1
ALT	Alanine transaminase
AST	Aspartate transaminase
FC	Fold change
OPLS-DA	Orthogonal partial least squares-discriminant analysis
PCA	Principal component analysis
